# Drinking alcohol raises the chance of premarital sex by four folds among secondary school adolescent students in Jima Arjo, Southwestern Ethiopia, 2018: a school-based cross-sectional study

**DOI:** 10.1186/s40834-022-00171-2

**Published:** 2022-04-12

**Authors:** Bayisa Biratu, Sileshi Garoma, Motuma Getachew, Markos Desalegn

**Affiliations:** 1 Family health department, East Wollega Zonal health office, Oromia region, West Nekemte, Ethiopia; 2 Department of Public health, Adama Medical College, Adama Central, Oromia region Adama, Ethiopia; 3 Department of Public health, Institute of Health Science, Wollega University, Oromia region, West Nekemte, Ethiopia

**Keywords:** Premarital sex, Drinking alcohol, Adolescent, Students

## Abstract

**Background:**

Premarital sexual practice is sexual intercourse performed before formal marriage. Pre-marital sexual practice increases adolescents’ risk for having multiple sexual partners, (sexually transmitted disease) STDs, and unintended pregnancy.

**Objective:**

The study aimed to assess the prevalence of premarital sexual practice and associated factors among secondary school (9–12 grade) students in the Jima Arjo district.

**Methods:**

Institutional-based cross-sectional study design was conducted among adolescent students from May 1st to 15th using a pre-tested and structured questionnaire. A systematic random sampling technique was employed to select study participants. The collected data was cleaned and entered into(statistical package for social science) SPSS version 20. Factors associated with the pre-marital sexual practice were identified by multiple logistic regression analyses.

**Results:**

The over all premarital sexual practice in this study area was 24.4%. Being alcoholdrinker(AOR[95%CI] = 3.78[1.49,22.08]),havingaboy/girlfriend(AOR[95%CI] = 5.07[3.74,26.47]), (being male) (AOR[95%CI] = 2.9[1.78,37.8]); urban residence (AOR[95%CI] = 6.44 [1.95,25.84]).

**Conclusions:**

The study revealed that a significant proportion of adolescent students in this study area practiced premarital sex. Being male sex, urban residence, using alcohol use and having a boy/girlfriend significantly affect premarital sexual practice. Therefore, school and community-based sexual health education, and communication need to be intensified to reduce premarital sex and further health consequences.

## Background

World Health Organization (WHO) defines adolescent people as those between the ages of 10 to 19 years [1]. Adolescence is the period of transition from childhood to adulthood at which young people experience significant physiological, psychological, and social changes following puberty; but do not immediately assume the roles and responsibilities of adulthood [2, 3].

These segments of the population account for one-fifth of the total world population and the majority(84%) of them are living in low and middle-income countries [4]. Ethiopia is one of the top ten countries with the greatest number of young populations [5].

Premarital sex is sexual intercourse performed between couples before marriage [5]. Globally sexual activities among adolescents have been reported to be increasing and Sub-Saharan Africa too [6]. In Ethiopia, the proportion of adolescents engaged in pre-marital sex ranges from 15 to 65% based on evidence from a study conducted in different parts of the country [7–12].

Evidence suggested that premarital sexual intercourse with multiple sexual partners has major problems on subsequent marital sexual satisfaction related to physiological and emotional characteristics which could result in divorce [13, 14].

Adolescent living in single-parent or with stepparents initiates sexual activity earlier than those in two-parent families whereas, feelings of closeness and connectedness to parents, parental disapproval of sex, and positive peer influences have been shown to delay sexual activity [15].

Abstinence from sex before marriage, delay of a sexual debut, and condom use are the major strategies used to reduce the spread of HIV infection in Ethiopia. However, school-based studies revealed that the prevalence of the premarital sexual practice and unprotected sexual practice is yet a common threat among school adolescents. Study on school adolescent premarital sex is not commonly conducted at periphery(district) level [8]. And also adolescents receive inadequate education, guidance, and services on reproductive health and this may lead them to practice premarital sex [16].

Early sexual initiation before marriage could be the reason to have multiple sexual partners which increase the chance of acquiring and transmitting sexually transmitted disease including HIV/AIDS [17]. Because consistent condom use is less among young people practicing sex at their early age [15]. Not only this but also unintended pregnancy is a serious problem of teenagers or adolescents practicing unsafe sex where 60% of adolescents’ pregnancy is unwanted or unintended resulted from unprotected sexual intercourse in Ethiopia [13, 18]. This could be the reason for not declining AIDS-related death and new HIV infections among adolescents [19]. Therefore this study was very important to identify the magnitude of premarital sex and associated factors among school adolescents in this study area.

## Methods

### Study area and period

The study was conducted in Jima Arjo district high school (9-12th grade) students, East Wollega Zone, Western Ethiopia from 1st to 15th of May 2018 GC. Jima Arjo district is one of the seventeenth districts of East Wollega Zone located at 379 Km to the west of Addis Ababa in the western part of Ethiopia and 48 km from Nekemte town, the capital city, of East Wollega Zone. The district has an estimated total population of 114,175 of which 55,946 were males and 58,229 were females. There were 3676 high school students (1903 males and 1773 females). The district has three public high schools, twenty Health posts, four Health Centers, one public hospital, and twelve private clinics.

### Study participants

The institutional-based quantitative cross-sectional study design was conducted among Jima Arjo District secondary school students. The Source population of this study was all 9th -12th grade secondary school students attending regular class in the 2018 GC academic year, in the Jima Arjo district and the study population was selected students from 9th -12th -grade secondary school students attending regular class during the study period.

All students aged 15 to 19 years, and attending regular class at the time of data collection were included. Those sick and unable to respond to the questionnaire were excluded from the study.

### Sample size determination

The required sample size was calculated by single population proportion formula using the following assumptions: the proportion of premarital sex among Jima school adolescents (25%) [9]; 95% confidence level, 5% precision between a sample and population parameter, and 10% possible none response rate.

$$n=\frac{\left(Z\partial/2\right)^2\;\mathrm p\;\left(1-\mathrm p\right)}{\mathbf d\mathbf2}$$

Where, 

**Z** = the z-score associated with selected degree of confidence interval at 95% confidence Interval is 1.96.

** P** = estimated percentage based on past study, 25% (0.25),d = margin of error 5%(0.05).

** n** = sample size.

Computed with the above formula **n = 288** and **10%** non respondense rate given a total sample size of **317**.

### Sampling Technique

First, the three high schools, Arjo, Gombo, and H/kumba were included in the study. Then students were classified according to their schools. Then stratified based on their grades (9th, 10th, 11th, and 12th). And sample was proportionally allocated to the size of each grade and. Finally, a simple random sampling technique was employed to select the participants using their roster as a sampling frame and their Id. The number was used to select individuals participants using a computer-generated random number.

### Data collection tool and procedures

The structured self-administered questionnaire was used to collect information from secondary school students in the Jima Arjo district. It was prepared in English and translated to Afan Oromo (the local Language) by an expert of both languages and was translated back to English to ensure its consistency. Pretest was done at Getema secondary school and a necessary amendment was taken before using the tool for actual data collection. Four data collectors were involved in data collection, and they were trained on how to approach respondents, the contents of the tool, and the objectives of the study. In addition, to the principal investigator, two supervisors were assigned to assist data collectors to check for completeness and consistency of a questionnaire.

## Operational definitions

### Adolescents

An individuals who are between the ages of 10–19 years old.

### Premarital sexual practice

Sexual intercourse performed before formal marriage.

### Age of sexual debut

The age at which the first sexual initiation occurred.

### Early sexual debut

Defined as having sexual intercourse before the age of 18 years.

### Variables of the study

#### Dependent variables

Premarital sexual practise.

#### Independent variables

Socio-demographic variables: (Age, sex, residence, grade level, Living arrangement, parental education, and marital status of parents.

Socio-economic factors: family monthly income and Pocket money of students.

Sexual history and reasons to start sexual practice: Having (boy/girlfriends), fall in love, sexual desire, peer pressure, money/Gift, Watching Pornographic film/sex movies, and rape.

Substance abuse: (alcohol consumption, chewing khat, smoking cigarette, and shisha).

### Data processing and analysis

After data collection, data were checked manually for completeness and consistency. It was sorted, entered, cleaned, and processed by SPSS version 20. Descriptive statistics were done to compute frequency; percentage, mean and median based on the type of data. Binary logistic regression was conducted and COR, with 95% CI was estimated to select the candidate variables for the final model. Then, variables with a *p*-value of < 0.25 at binary logistic regression were taken into a multivariable logistic regression to control confounding. Hosmer-Lemeshow goodness-of-fit with stepwise (backward elimination) logistic regression was used to test for model fitness. AOR with 95% CI was estimated to assess the presence of association at multivariable logistic regression. Finally, variables with a *p*-value of < 0.05 were considered as statistically significant predictors of the outcome variable.

### Data Quality management

The quality of data was assured by properly designed and pre-tested questionnaires, trained data collectors, and supervisors for data collection procedures. The Instrument was pretested on 5% of the respondents at Getema high school and amendment was taken accordingly. Every day, the collected questionnaires were reviewed and checked for completeness by the supervisors and principal investigator. The necessary feedback was offered to data collectors before the actual procedure. Data quality was also ensured by coding, cleaning, and double-entry of at least 5% of the questionnaires.

## Results

### Socio-demographic characteristics of adolescent students in Jima Arjo district

A total of 312 students participated in the study, making a response rate of (98.4%). Among the respondents, 170 (54.5%) were males in sex, and their ages ranged from 14 to 19 years with a mean age of 17.29 (± SD1.10) years. Most of them, 238 (76.3%) were grade 9th − 10th. The majority of the participants, 280 (89.7%), were Oromo and the remaining 32 (10.3%) were Amara.

By religion, 161(51.6%) were Protestant, 41(45.2%) were Orthodox and the rest 10(3.2%) were Muslims. The majority of them, 190(60.9%) were rural and 122 (39.1%) urban residents (Table 1).

**Table 1:** Socio demographic characteristics of secondary school adolescent students (9-12th grade) in Jima Arjo district 2018.

Variables	Characteristics	Frequency	Percent
Age	10-14years	4	1.3
	15-19years	308	98.7
	Total	312	100
Sex	Male	170	54.5
	Female	142	45.5
	Total	312	100.0
Grade level	9th	135	43.3
	10th	103	33.0
	11th	41	13.1
	12th	33	10.6
	Total	312	100.0
Current residence	Urban	122	39.1
	Rural	190	60.9
	Total	312	100
Parent marital status	Married	270	86.5
	Divorced	32	10.3
	Widowed	10	3.2
	Total	312	100
Pocket money of students	Yes	156	50
	No	156	50
	Total	312	100

#### Premarital sex and other sexual characteristics of adolescent students in Jima Arjo district

Among all adolescent students who took part in the study, 213(68.3%) had no boy/girlfriends the rest 99 (31.7%) had boy/girlfriends. Seventy-six (24.35%) of them responded that they experienced premarital sexual intercourse; 44(14.10%) males and 32(10.25%) females respectively.

**Fig. 1:**
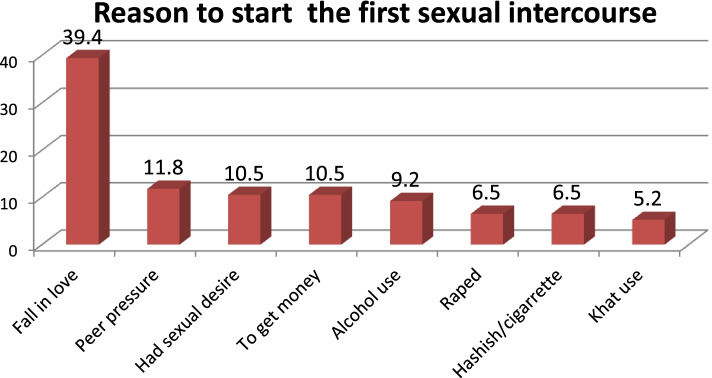
Reason to start the first sexual intercourse among secondary school (9th -12th grade) adolescent students in Jimma Arjo district, may, 2018

The minimum age at the first sexual practice of participants was 13 and the maximum age was 19 years. The main reasons reported by respondents for the sexual debut were falling in love(39.4%) followed by peer pressure(11.8%) (Fig. 1). The majority of those who experienced premarital sexual intercourse 59(77.6%) had 1 sexual partner,17(22%) have > 2 partners. The majority of the respondents had boy/girlfriends 45 (59.2%) (Table 2).

**Table 2:** Sexual history and reasons to start first sexual intercourse of secondary adolescents students(9th-12th grade) of Jimma Arjo district may,2018

Variables	Characteristics	Frequency	Percent
Had boy/girl friend of sexual partner	Yes	99	31.7
	No	213	68.3
	Total	312	100.0
Ever had sexual intercourse	Yes	76	24.4
	No	236	75.6
	Total	312	100.0
Number of your sexual partner	One	59	77.6
	Two	12	15.8
	Three	5	6.6
	Total	76	100
Age at first sexual intercourse	10-13years	1	1.3
	14-16years	45	59.2
	17-19years	30	39.5
	Total	76	100.0
Age of partner at first sexual intercourse	10-13years	5	6.6
	14–16 years	27	35.5
	17–19 years	32	42.1
	> 19 years	12	15.8
	Total	76	100.0

**Table 3:** Unprotected/protected sexual practices among secondary school adolescent students in Jimma Arjo district, 2018

Variables	Characteristics	Frequency	Percent
Sexual intercourse in the last 12months	Yes	67	88.1
	No	9	11.8
	Total	76	100
Number of your sexual partner in last 12 m	One	51	76.1
	Two	13	19.4
	Three	3	4.4
	Total	67	100
For males sexual intercourse in last 12 m with	Non Commercial sex worker	36	87.8
	Commercial sex worker	1	2.4
	Both	4	9.7
	Total	41	100
Condom use in last sexual intercourse	Yes	29	38.1
	NO	47	61.8
	Total	67	100
Suggestion given(Initiator for condom use)	My self	5	17.2
	My partner	2	6.8
	By joint	22	75.8
	Total	29	100
Reason to use condom	For fear of HIV/AIDS	15	51.8
	To prevent pregnancy	10	34.4
	To prevent STIs	4	13.8
	Total	29	100

#### Substance use and other sexual behaviors of adolescent students in Jima Arjo district

About twenty-six percent of the respondents, 80(25.6%) reported that they use a substance like alcohol, khat, and cigarette/shisha. Fifty-one (16.3%) of them use alcohol, 17(5.4%) use Khat, 12(3.8%) smoke cigarettes or shisha.

Among the study participants who have experienced premarital sex(76 students), a larger proportion, 67(88.2%) of them had sexual intercourse 12 months before the survey.

Concerning the total number of partners, they have had in the past 12 months; 51(76.1%) of them had sex with only one partner, 13(19.4%) had sex with two partners and the rest 3(4.4%) had had sex with over two partners.

The majority, 47(61.8%) of the respondents in this study area did not use a condom during their last sexual intercourse. The main reason mentioned by respondents for not using a condom was: didn’t know how to use 14(29.6%), ashamed to ask partner 13(27.6%), ashamed to buy condom 6(12.7%), trust their partner 5(10.6%), decreased their sexual satisfaction 3(6.3%) and the rest 3(6.3%) of them were not using a condom because the condom was not easily available(Table 3).

#### Factors associated with premarital sexual practice among adolescent students

Socio-demographic variables, sex of the respondents, Ethnic group of the respondents, the residence of the respondents, Grade level of the respondents, parent marital status of the respondents, and from the socioeconomic variables monthly income of family and pocket money of the respondents were evaluated using logistic regression against the premarital sexual practice.

In the final multivariate logistic regression model; sex, residence, substance use/alcohol use, and having a girl/boyfriend were statistically significant to affect respondents premarital sexual practice. Male adolescents were more likely to be involved in premarital sexual experience than females (AOR [95%CI] = 2.9[1.78,37.8]). Urban residents were more likely to involve in premarital sex than rural (AOR[95%CI] = 6.44[1.95,25.84]). Students of grade 9th had 93% lesser odds of practicing premarital sex compared to grade 12th-grade students (AOR[95%CI] = 0.07[0.01,0.81]). Adolescents who drink alcohol were about four times more likely to practice premarital sex compared to respondents who did not use alcohol (AOR[95%CI] = 3.78[1.49,22.08]). Respondents who had a boy/girlfriend were 5.08 times more likely to practice premarital sex compared to their counterparts (AOR [95%CI] = 5.07[3.74,26.47]).

But Ethnicity, Parent marital status, having pocket money, Parent monthly income, cigarette/hashish use did not show statically significant association with premarital sexual intercourse among high school adolescent students in the Jimma Arjo district (Table 4).

**Table 4:** Multi variate and bivariate analysis of selected variables affecting premarital sexual practice among high school Adolescents students, Jimma Arjo, 2018

Variables	Premarita lsexual practice		COR (95% CI)	AOR (95% CI)
	**No (%)**	**Yes (%)**		
Grade level				
Grade 9th	106(78.5)	29 (21.5)	3.88 (1.75, 8.61**)**	*0.07(0.01,0.81)
Grade 10th	85 (82.5)	18(17.5)	5.01 (2.14, 11.75)	0.87(0.08,8.81)
Grade 11th	29 (70.7)	12 (29.3)	2.56 (0.98, 6.69)	0.24(0.01,3.23)
Grade 12th	16 (48.5)	17 (51.5)	1.00	1.00
Sex				
Male	126(74.1)	44 (25.9)	0.83 (0.49, 1.40)	*2.9(1.78,37.78)
Female	110 (77.5)	32 (22.5)	1.00	1.00
Residence				
Urban	81(66.4)	41(33.6)	0.44 (0.26, 0.75)	*6.44(1.95,25.84)
Rural	155(81.6)	35 (18.4)	1.00	**1.00**
Having Boy/Girl friend Yes	33 (33.3)	66 (66.7)	0.02 (0.01, 0.05)	*5.07(3.74,26.47)
No	203 (95.3)	10 (4.7)	1.00	1.00
Use alcohol				
Yes	5(9.8)	46(90.2)	0.01 (0.01, 0.03)	*3.78(1.49,22.08)
No	231(88.5)	30 (11.5)	1.00	1.00
Parent monthly income in birr				
smoke cigarette/shisha				
Yes	1 (8.3)	11 (91.7)	0.02 (0.003, 0.19)	0.06(0.00,7.63)
No	235 (78.3)	65 (21.7)	1.00	1.00

## Discussion

In this study, 24.4% of the adolescent students had premarital sex. This is in line with the finding from a study conducted in Jima preparatory adolescent and Agaro high school adolescent students which revealed premarital sex of 25% each [9, 20]. But lower than a study in Sebeta secondary school (9–12 grade) adolescent students, 28.4% [16], southeast Ethiopia(31.2%) [6], southwest Ethiopia (43.8%) [9], and northwest Ethiopia(60.2%) [8]. This could be due to participants of Jima Arjo are less susceptible to the effect of globalization because they are far from the capital Addis Ababa and they may have less access to sexually explicit materials like pornographic films which could lead them to practice premarital sex. Another possible explanation could be because northwest Ethiopia, Debra tabor town, was a zonal town having higher education institutions like Debra Tabor University which could increase the number of young populations with increased demand for sexual practice. The finding from southwest Ethiopia has a different study setting from this study which was conducted in Rift Valley University.

Premarital sex in this study area was higher than that of Nekemte town (21.4%) [4], Alamat 21.1% [21], Ambo high school adolescent student19.4% [22], North Gojjam 19% [8], and Sidama zone 18% [7]. This might be due to the variation of study area or study period(2010–2016), which shows changing and improving trend in reporting premarital sex or globalization which tend to increase access to social media ann other sexually explicit material which could have a role in increasing chance of premarital sex in this study area.

In the multivariable logistic regression model, Sex of the respondent has an association with premarital sexual practice. Males were about three times more likely to practice premarital sex than females. The finding of this study was in line with Gedeo zone high school adolescents [7], Nekemte town high school adolescent students [4], Ashendi town west Gojjam [8], Ambo high school adolescents students [22], Balegoba [23], Wollega University [24].

The residence of the respondents was also associated with premarital sexual practice. The adolescent students from urban were 6.44 times more likely to be engaged in pre-marital sexual practice than their counterparts. This finding is consistent with the study conducted in Alamata high school adolescent students [25], Jimma town school adolescent students [9], and Eastern Ethiopia [26]. This could be due to liberal lifestyles in urban areas compared to cultural conservatism in rural areas, and may also be attributed to the easiness of reporting pre-marital sex by urban adolescents. The urban environment provides a conducive environment for experimenting with sex before marriage and adolescent students in urban get access to sexually explicit materials which could increase the chance of practicing premarital sex [12, 24, 26].

The grade level or educational level of the student had an association with the prevalence of premarital sexual practice which is consistent with the study conducted in Sebeta high school adolescents [16] and in Eastern Ethiopia [26]. The most likely explanation might be; as the grade level of grade increase chance of departure from parents increase, parental control gets weaken, which leads them to engage in premarital sexual practice.

Also, as their grade level increase, age increase, which in turn increase premarital sex [21].The adolescent student who uses alcohol was about four times more likely to practice premarital sex. This was consistent with a study conducted in Jimma town school adolescents [9], North East Ethiopia high school adolescent students [12], West Shewa Zone school adolescent students [22], Mada Walabu university [23], Jimma zone school adolescent [28]. The reason might be, drinking alcohol decrease self-control and predispose to risky sexual behavior such as unplanned, unprotected sexual intercourse including premarital sex.

Adolescents who have a boy/girlfriends were about 5 times more likely to involve in premarital sexual practices than those who do not have a boy/girlfriend and consistent with a study done in Alamata high school adolescent students [25], Nekemte high school adolescent students [4], and Jigjiga University students [29]. This could be because of the pressure from their girl/boyfriend to have a sexual practice which is the second common reason (11.8%) why adolescents practiced premarital sex.

The study has different limitations. First; the study used a cross-sectional study design that cannot establish trends and causality between premarital sex and risk factors. Second, the data was collected using a self-administered questionnaire which may be subjected to recall bias and under-reporting of premarital sex among respondents because of social desirability bias. Finally, the study was school-based which might have missed the adolescent not attending the school. Despite the limitations, this study has valuable finding which encourages the family, community, teachers and other stakeholders to give attention for their adolescent students.

## Conclusions

The study revealed that a significant proportion of adolescent students in this study area practiced premarital sex. Being male in sex, urban residence, having boy/girlfriends, and drinking alcohol were found to affect adolescent students’ premarital sexual practice. Therefore, families, the community, and the School should closely communicate with adolescent students about their sexuality and problem behaviors putting them at risk of premarital sex or further health consequences. Those stakeholders should be transparent and provide adolescent students with health education, and communication-related to sexual and reproductive health in collaboration with local health professionals. Further qualitative research should be conducted to explore the concern of key informants and adolescents on premarital sex and its associated factors.

## Data Availability

The corresponding author can make the required data and material whenever needed.

## Abbreviations

AIDS: Acquired Immuno Deficiency Syndrome
AOR: Adjusted Odds Ratio
CI: Confidence Interval
COR: Crude Odds Ratio
HIV: Human Immunodeficiency Virus
RSB: Risky Sexual Behaviour
STD: Sexually Transmitted Disease
WHO: World Health Organization
